# Supplementary material for: The ACC-Deaminase Producing Bacterium Variovorax sp. CT7.15 as a Tool for Improving Calicotome villosa Nodulation and Growth in Arid Regions of Tunisia
Source: Microorganisms. 2020 Apr 9;8(4):541. doi: 10.3390/microorganisms8040541 (PMC7232455; doi:10.3390/microorganisms8040541)
Supplement: Supplementary file 1 [file microorganisms-08-00541-s001.pdf]

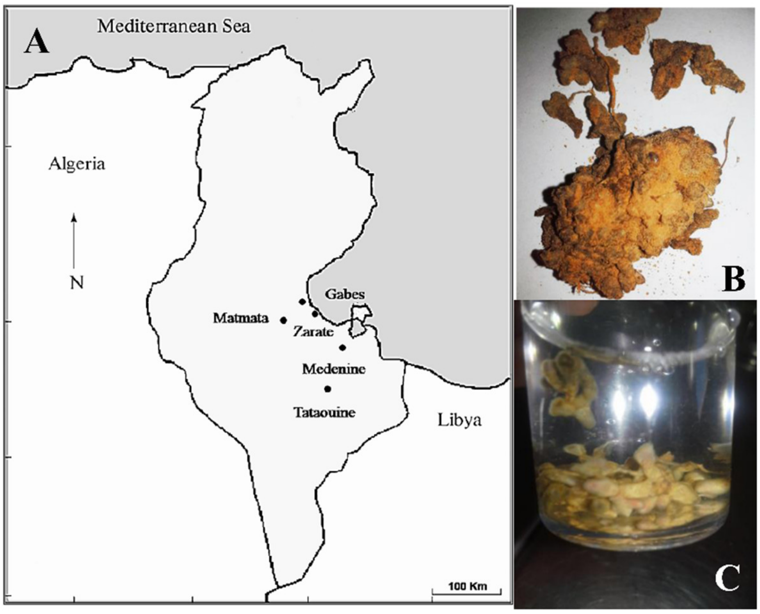


**Figure S1.** (A) Map of Tunisia showing the location of sampling sites. (B) and (C) Nodules of *Calicotome villosa* wild type plants collected for this work.

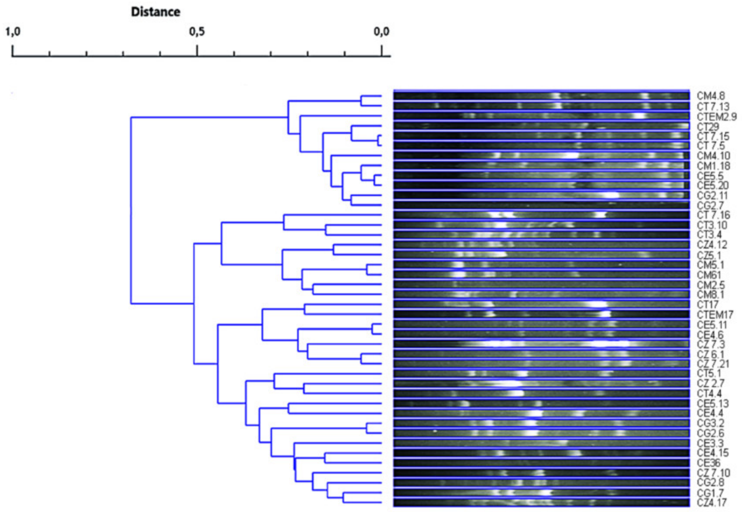


**Figure S2.** Box-PCR patterns of the bacteria isolates from *Calicotome villosa* compared using dendrogram. Scales at the top of the dendrograms represent similarity.

**Table S1.** Cross-nodulation of representative rhizobia isolated from *C. villosa* nodules with some grain legumes.

| Strain | Related species | *Medicago sativa* | *Lens culinaris* | *Lotus edulis* |
|---|---|---|---|---|
| CZ4.17 | *Neorhizobium galegea* | - | - | - |
| CZ7.10 | *N. galegea* | + | - | - |
| CZ5.1 | *N. galegea* | + | - | - |
| CZ2.7 | *Rhizobium sullae* | + | + | + |
| CT4.4 | *R. sullae* | + | + | - |
| CE5.13 | *R. sullae* | + | + | + |
| CTEM17 | *Phyllobacterium ifriqiyense* | - | - | - |

| | | | | |
|---|---|---|---|---|
| CE5.11 | *P. ifriqiyense* | - | - | - |
| CT7.16 | *P. ifriqiyense* | - | - | - |
| CE36 | *Rhizobium pakistanense* | + | - | - |
| CZ7.21 | *R. pakistanense* | - | - | - |
| CM61 | *Ensifer meliloti* | + | + | + |
| CM8.1 | *E. meliloti* | + | + | + |
| CM2.5 | *E. meliloti* | + | + | + |
